# Incidentally Detected Large Choroidal Melanoma in a 27-Year-Old Asymptomatic Woman: A Case Report

**DOI:** 10.7759/cureus.113395

**Published:** 2026-07-26

**Authors:** Oussama Benhar, Ayoub Lekhlifi, Zakaria Azemour, Nabil Bouslous, Moustaine Omar

**Affiliations:** 1 Ophthalmology, Université Ibn Zohr, Agadir, MAR; 2 Ophthalmology, Centre Hospitalier Universitaire Mohammed VI, Agadir, MAR

**Keywords:** choroidal malignant melanoma, melanoma, ocular tumor, primary melanoma, young adult males

## Abstract

Choroidal melanoma is the most common primary intraocular malignancy. Although rare, early diagnosis is crucial given its significant impact on both visual and vital prognoses. It often develops insidiously and is discovered incidentally. We report the case of a 27-year-old asymptomatic woman in whom a choroidal melanoma was incidentally discovered during a routine ophthalmologic examination. Fundus evaluation revealed a large inferotemporal pigmented choroidal mass in the left eye. Multimodal imaging, that is, B-scan ultrasonography, optical coherence tomography, fluorescein and indocyanine green angiography, and orbital magnetic resonance imaging (MRI), documented a characteristic mushroom-shaped lesion measuring 9.42 mm in largest basal diameter and 10.74 mm in apical thickness, with low-to-medium internal reflectivity, associated exudative retinal detachment, and a T1-hyperintense/T2-hypointense signal pattern. A contrast-enhanced whole-body computed tomography (CT) scan was performed, revealing two solid pulmonary parenchymal nodules in the right lower lobe, measuring 5 mm and 4 mm. Given the tumor dimensions, location, and extension, globe-conserving brachytherapy was not feasible, and urgent enucleation was performed. Management of choroidal melanoma ranges from globe-conserving radiotherapy to enucleation, with prognosis closely tied to tumor size at diagnosis and to the risk of hematogenous metastasis. The educational message of this case is twofold: choroidal melanoma may occur in young, asymptomatic patients and remain clinically silent until an advanced stage, and systematic dilated fundus examination, even in low-risk populations undergoing routine refraction, remains the single most effective means of detecting a potentially lethal intraocular malignancy at a treatable stage.

## Introduction

The choroid can be the site of a wide variety of malignant and benign, primary and secondary tumors. Choroidal melanoma is the most common primary intraocular malignancy, yet it remains a rare disease, with an incidence of 5-9 cases per million inhabitants per year [1]. It is predominantly a tumor of the sixth and seventh decades, with a median age at diagnosis of approximately 60 years. Presentation before the age of 30 is distinctly uncommon, accounting for less than 1% of all cases, and pediatric or young-adult uveal melanoma is often reported in association with predisposing conditions such as oculodermal melanocytosis, familial BAP1 tumor predisposition syndrome, or dysplastic nevus syndrome [2,3].

This age-related rarity carries direct clinical consequences. In young adults, a pigmented choroidal lesion is far more likely to be interpreted as a nevus or a benign melanocytic proliferation, and the absence of the usual demographic context may delay referral to an ocular oncology unit. The problem is compounded when the tumor is entirely asymptomatic, as peripheral lesions sparing the macula frequently are, so that detection depends solely on systematic dilated fundus examination.

Choroidal melanoma is a severe condition that compromises both the visual prognosis of the affected eye and the patient's overall survival, with mortality driven by hematogenous metastatic dissemination, principally hepatic and pulmonary. Advances in conservative treatment, particularly plaque brachytherapy and proton beam radiotherapy, now allow globe preservation in most cases, enucleation being reserved for large tumors, those with extrascleral extension, or those with optic nerve involvement [4]. Because eligibility for conservative management is dictated largely by tumor dimensions at presentation, early diagnosis is crucial and relies on recognizing suspicious lesions and distinguishing them from other pigmented choroidal lesions.

The present case adds to the limited literature on choroidal melanoma in very young adults in three respects: it documents a large, advanced tumor already associated with suspicious pulmonary nodules in a completely asymptomatic 27-year-old woman with no identifiable predisposing syndrome; it illustrates the full multimodal imaging phenotype of such a lesion at this age; and it demonstrates that the window for globe-conserving therapy may already be closed at the time of an incidental first diagnosis, reinforcing the argument for systematic peripheral fundus examination in young patients presenting for routine refraction.

## Case presentation

We report the case of a 27-year-old woman with no significant medical or traumatic history who presented to our clinic for a routine ophthalmologic examination without any disturbing symptoms.

On ophthalmologic examination, visual acuity was preserved at 10/10 in both eyes. Examination of the anterior segment was normal bilaterally, with a clear cornea, a deep and quiet anterior chamber, and a transparent crystalline lens. Intraocular pressure was measured at 13 mmHg in both eyes.

**Figure 1:**
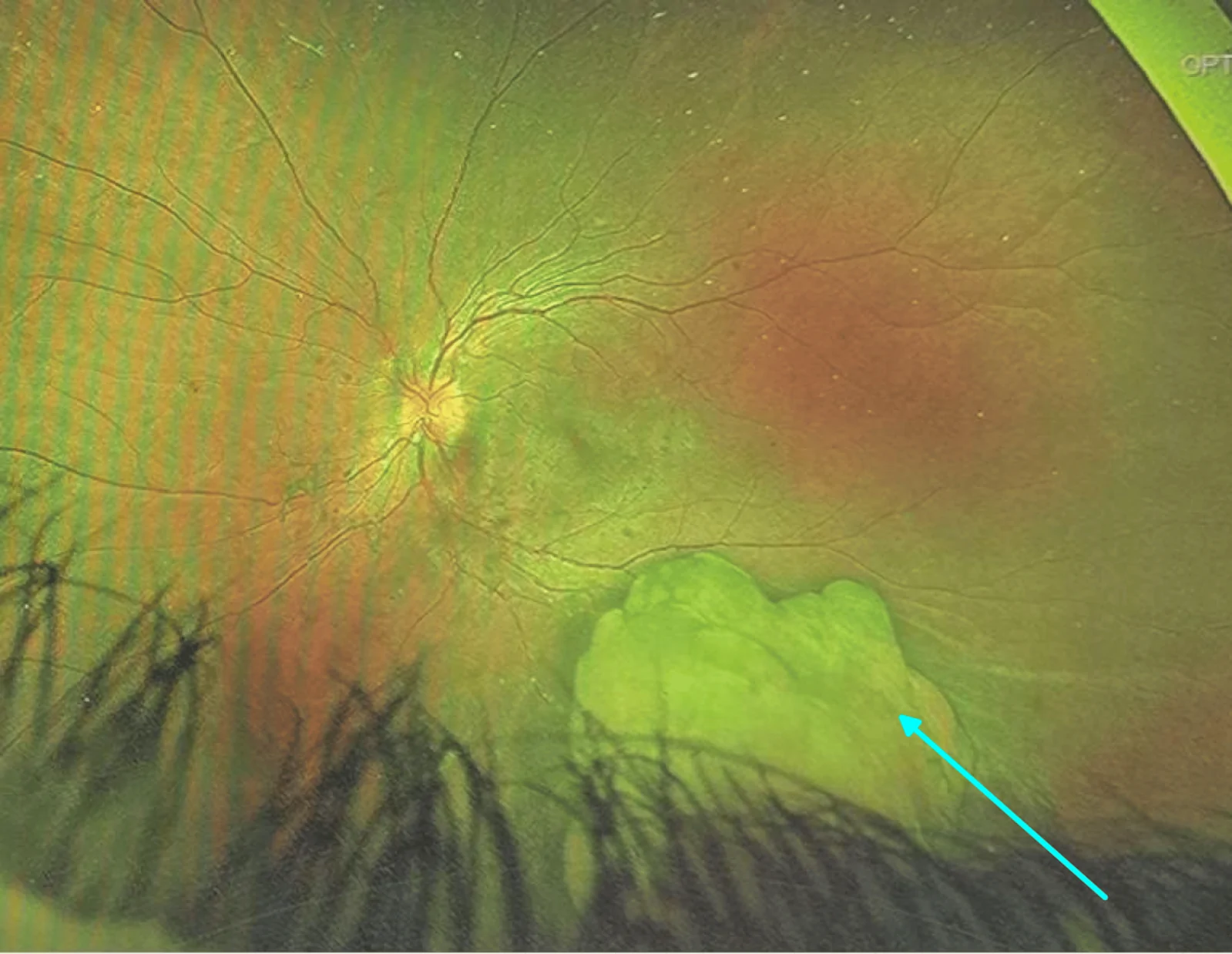
Fundus photography of the left eye revealing a large, polylobulated, budding choroidal mass located in the inferotemporal region (blue arrow), associated with optic disc edema

Fundus examination of the left eye revealed a large, polylobulated, budding choroidal mass located inferotemporally, associated with multiple pigmented spots at the posterior pole, vitreous pigment dispersion, and stage 1 optic disc edema (Figure 1). Fundus examination of the right eye was unremarkable. Gonioscopy was normal in both eyes.

**Figure 2:**
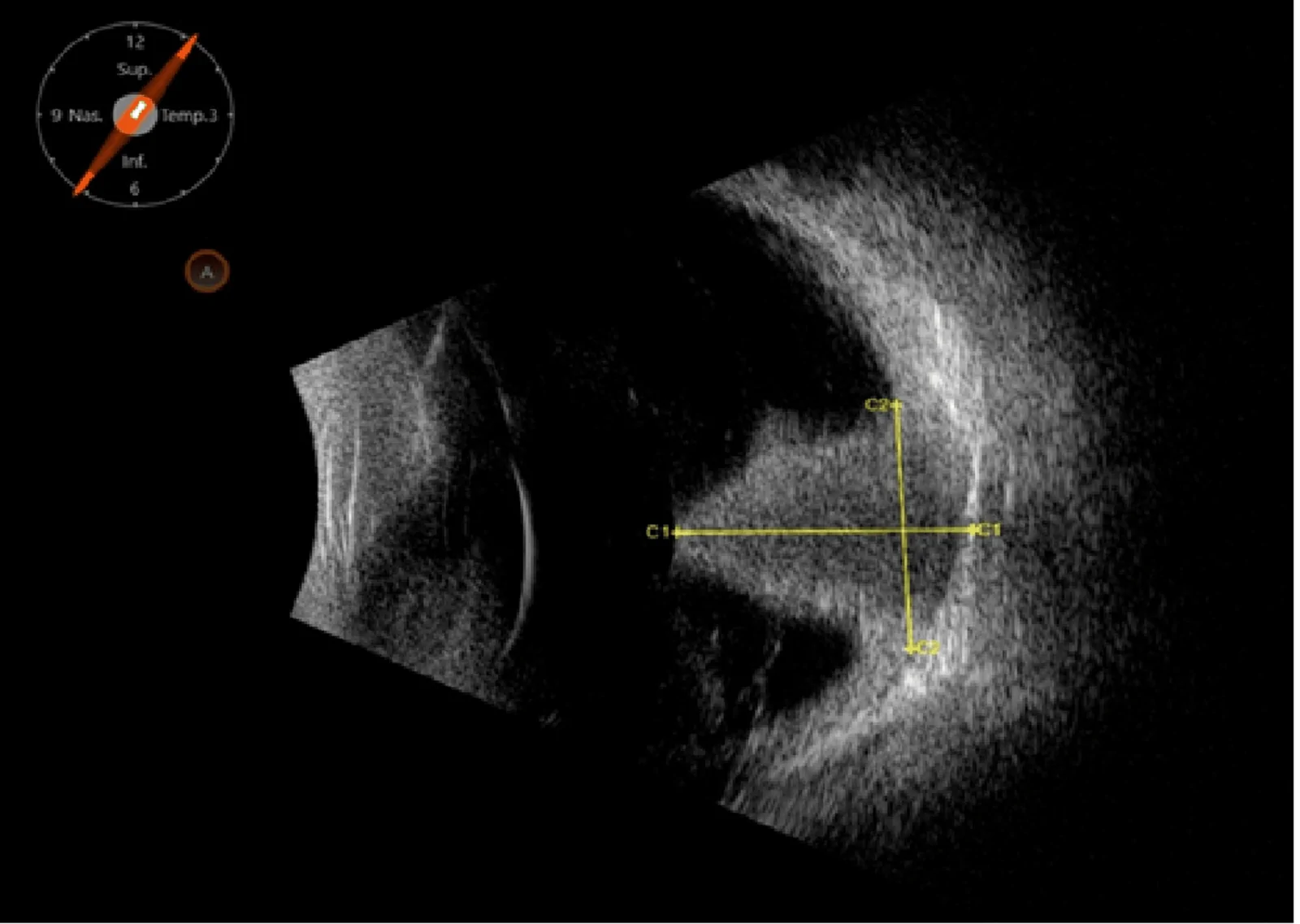
B-scan ultrasonography revealing a mushroom-shaped mass with central hyperechogenicity, with progressive hypoechogenicity toward the base

B-scan ultrasonography revealed a mushroom-shaped mass measuring 10.74 mm in apical thickness and 9.42 mm in basal diameter, which was hyperechoic centrally and became progressively hypoechoic toward its base (Figure 2).

**Figure 3:**
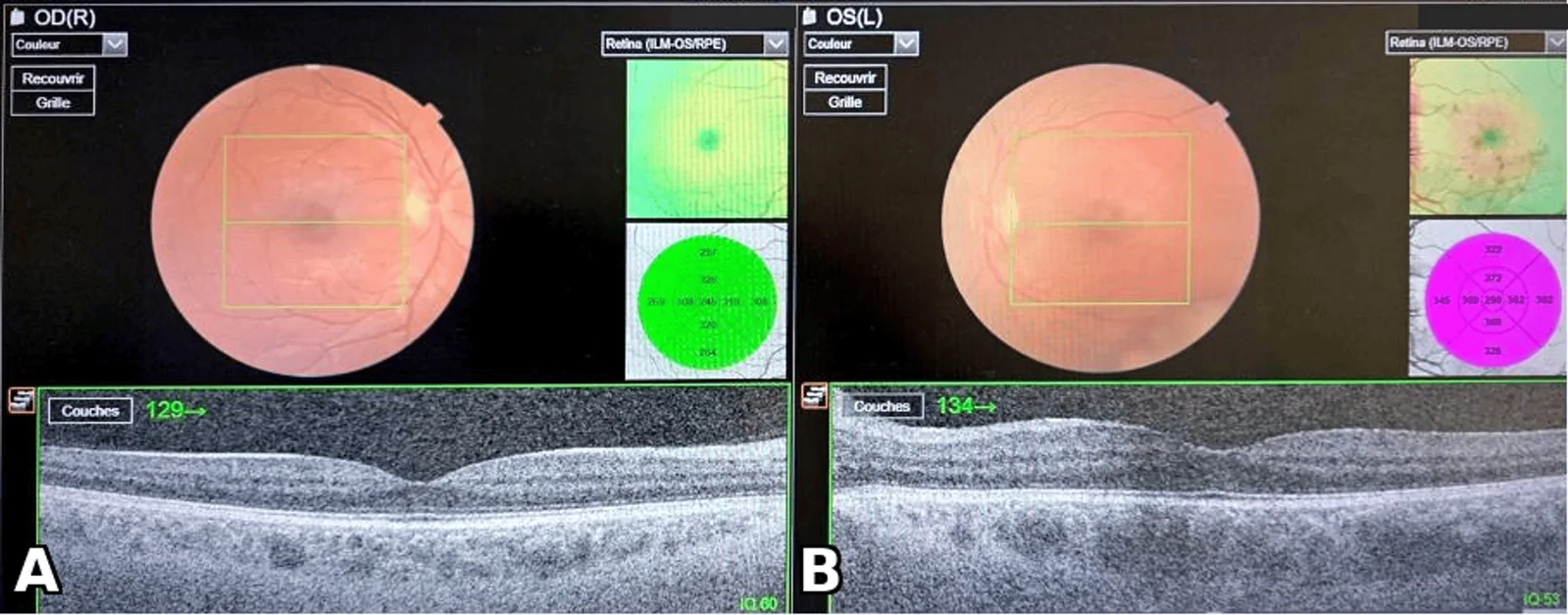
Macular optical coherence tomography of both eyes (right eye (A) and left eye (B)) demonstrating diffuse macular thickening in the left eye (B) with preservation of the foveal contour

Macular optical coherence tomography of the left eye showed diffuse macular thickening with preservation of the foveolar contour (Figure 3).

**Figure 4:**
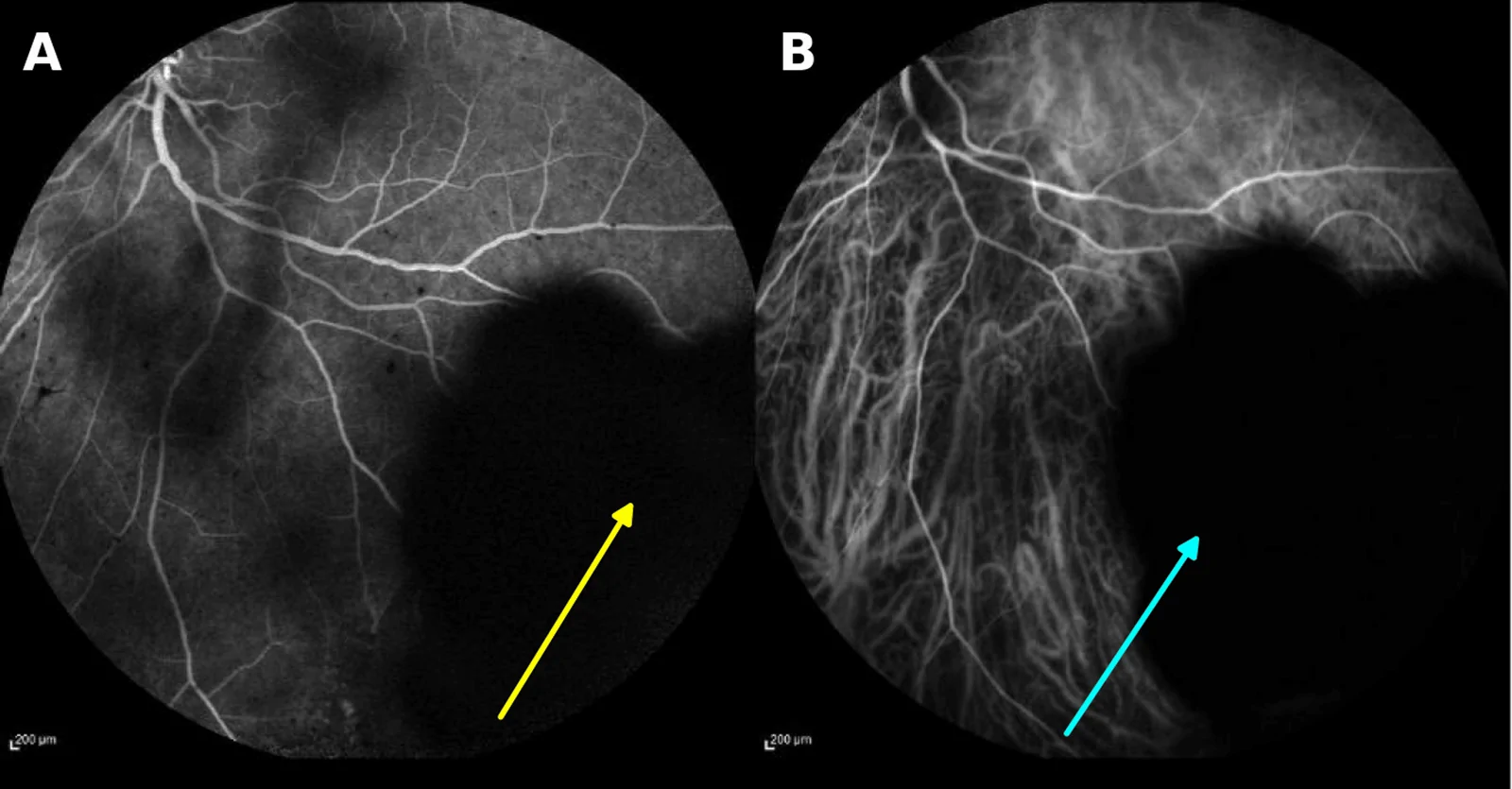
(A) Fluorescein angiography and (B) indocyanine green angiography demonstrating a markedly hypofluorescent, hypocyanescent lesion in the early phases (yellow and blue arrows), consistent with pigmentary masking

Fluorescein angiography and indocyanine green angiography were performed, demonstrating a lesion that was markedly hypofluorescent, hypocyanescent in the early phases, consistent with pigmentary masking (Figure 4).

**Figure 5:**
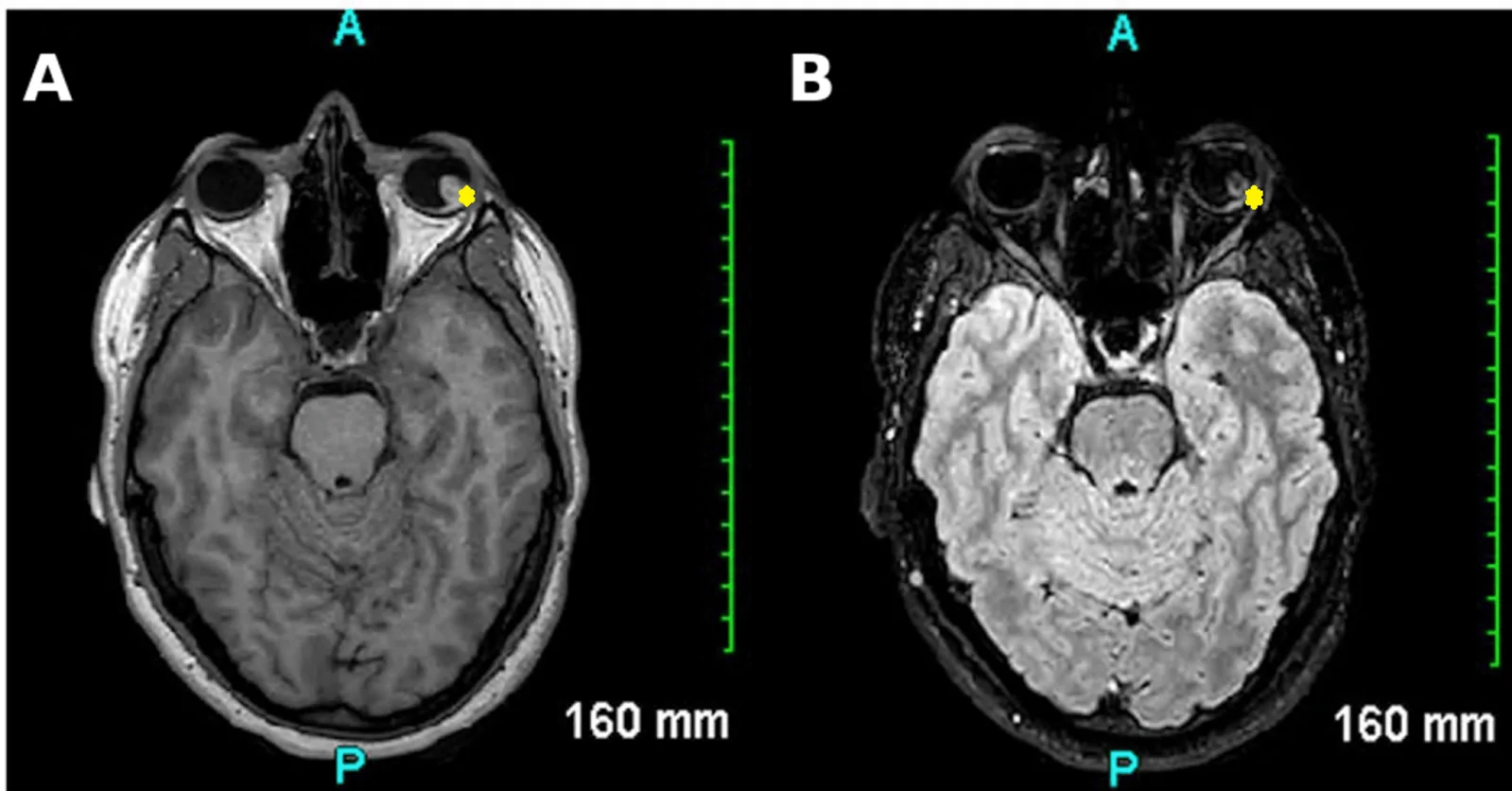
(A) T1-weighted and (B) T2-FLAIR orbital MRI showing a mushroom-shaped nodular lesion (yellow asterisk) located inferotemporally in the left eye, characterized by spontaneous T1 hyperintensity and T2 hypointensity, with peripheral enhancement and absence of central enhancement FLAIR: fluid-attenuated inversion recovery; MRI: magnetic resonance imaging

Orbital magnetic resonance imaging (MRI) demonstrated a mushroom-shaped nodular lesion on the inferotemporal region of the left eye, showing spontaneous axial T1-weighted hyperintensity and axial T2-weighted hypointensity with peripheral enhancement, without central contrast uptake (Figure 5). No other signal abnormalities were noted in the brain or in the contralateral orbit.

A contrast-enhanced whole-body computed tomography (CT) scan was performed, revealing two solid pulmonary parenchymal nodules in the right lower lobe, measuring 5 mm and 4 mm, considered suspicious in this context. No abnormal lesions were identified elsewhere, in particular no hepatic lesion, the liver being the preferential metastatic site of uveal melanoma. Given the sub-centimeter size of both nodules and the known variable fluorodeoxyglucose (FDG) avidity of uveal melanoma metastases, ¹⁸F-FDG positron emission tomography (PET)/CT was not considered contributory at this stage, and short-interval thoracic CT surveillance at three months was preferred. A medical oncology consultation has been scheduled, and PET/CT will be performed should the nodules demonstrate interval growth or should additional lesions appear. At the time of writing, definitive characterization of the pulmonary nodules is pending.

A diagnosis of choroidal melanoma was established on the basis of the clinical and multimodal imaging findings described above. Therapeutic management consisted of urgent enucleation of the left eye. The immediate postoperative course was marked by left frontal headache, nausea, and dizziness, unrelieved by paracetamol. Contrast-enhanced brain CT, performed to exclude cerebral venous thrombosis, intracranial hemorrhage, and infection, was unremarkable. These symptoms were therefore attributed to the immediate postoperative period following enucleation and resolved with conservative management within five days, with no bearing on the oncological course.

## Discussion

Uveal melanomas are the most frequently symptomatic ocular tumors in adults. They mainly involve the choroid (80%) [5]. Among the predisposing factors, the following may be cited: age over 55 years, fair skin and light-colored iris, UV exposure, a germline BAP1 mutation, ocular or oculopalpebral melanosis known as nevus of Ota, and the presence of cutaneous, iris, or choroidal nevi [2,6-8].

Choroidal nevi are pigmented lesions of the choroid present in approximately 6% of Caucasian individuals. Their malignant transformation has been estimated by Singh et al. at about one in 8,845 cases [9]. Their presence warrants annual monitoring for benign nevi, with closer and more tailored follow-up in cases with suspicious features or risk factors [2].

The development of choroidal melanoma is generally insidious and without obvious clinical signs. When present, symptoms are mainly visual. Reduced visual acuity, when present, reflects tumor extension to the posterior pole or associated macular edema. Visual field loss is indicative of a large tumor or associated retinal detachment. Myodesopsia may be observed in cases associated with retinal tears. Vitreous hemorrhage may also occur. Ocular pain has been reported in cases of tumor invasion of the ciliary nerves or secondary inflammation due to tumor necrosis. Angle-closure glaucoma may occur in the presence of a large anterior tumor.

However, choroidal melanoma is often discovered incidentally, accounting for approximately 30-40% of cases [1]. The diagnosis of choroidal melanoma is primarily based on dilated fundus examination. In its early stages, it typically appears as a dome-shaped oval lesion that may later develop a characteristic mushroom-like configuration, often associated with orange pigment corresponding to lipofuscin deposits, sometimes in a heterogeneous distribution. Pigmentation is variable, ranging from black to light brown in most cases, and some tumors may be completely amelanotic [2]. Documented tumor growth is a strong argument in favor of the lesion's malignancy [1].

Typical benign nevi generally do not present diagnostic difficulty. They are small in size (1-5 mm in diameter), flat or only slightly elevated, asymptomatic, and often dotted with drusen, which reflect their chronicity [10]. The situation becomes more challenging with suspicious nevi [11]. A nevus is considered suspicious when at least one of the following features is present: presence of recent visual disturbances (scotoma, metamorphopsia, myodesopsia, etc.), clinically visible serous retinal detachment, thickness greater than 2 mm, diameter greater than 7 mm, and presence of orange pigment on the surface or pinpoint hyperfluorescence on angiography.

In the presence of a suspicious nevus, closer follow-up is recommended: every three months for two years and then every six months. If documented growth occurs, the lesion is considered an early melanoma, and radiotherapy is initiated [10].

The differential diagnosis of choroidal melanoma includes several tumoral and pseudotumoral lesions of the fundus. Among benign pigmented lesions are optic disc melanocytoma, a peripapillary, often heavily pigmented and typically non-progressive lesion, as well as congenital hypertrophy of the retinal pigment epithelium. Combined hamartoma of the retina and retinal pigment epithelium is a rare, congenital benign tumor [12], presenting as a greyish elevated lesion associated with overlying vascular tortuosity [13]. The differential diagnosis also includes choroidal lesions such as choroidal hematomas, choroidal osteomas, and choroidal hemangiomas. Choroidal metastases may be confused with amelanotic forms. Idiopathic choroidal calcifications should also be considered. Certain tumors of the retinal pigment epithelium, such as adenoma and adenocarcinoma, may mimic melanoma. Finally, conditions such as posterior scleritis and uveal effusion syndrome should also be considered in the differential diagnosis [1].

In the case of a typical lesion, the clinical appearance is generally sufficient to establish the diagnosis. However, the following complementary examinations should be systematically performed: Color fundus photography, including red-free and blue-light imaging, provides a baseline photographic documentation. B-scan ultrasonography shows a lenticular, dome-shaped, or mushroom-shaped lesion. Choroidal melanoma markedly attenuates ultrasound waves, resulting in a hypoechoic appearance at the tumor base and a characteristic choroidal excavation [2]. It also allows essential measurement of tumor dimensions and the detection of extrascleral extension, which is an adverse prognostic sign and requires additional treatment. Autofluorescence imaging shows hypoautofluorescence corresponding to retinal pigment epithelium alterations and hyperautofluorescence related to orange pigment deposits. Fluorescein angiography shows a masking effect due to orange pigment and melanin migration. In the late phases, it reveals heterogeneous hyperfluorescence with pinpoint leaks, corresponding to focal disruptions of the retinal pigment epithelium. Indocyanine green angiography shows a markedly hypofluorescent lesion in the early phases, due to pigment masking, followed by visualization of intratumoral choroidal vasculature, which has prognostic value. Optical coherence tomography allows the visualization of serous retinal detachment, thickening of the outer retina overlying the lesion, cystoid spaces, and hyperreflective lipofuscin deposits.

Orbital and cerebral MRI is part of the work-up for locoregional extension, particularly in cases of optic disc involvement, allowing the assessment of scleral integrity and invasion of the optic nerve head. Intraocular melanomas are typically hyperintense relative to the vitreous on T1-weighted images and hypointense on T2-weighted images and show contrast enhancement [4].

The initial staging work-up for choroidal melanoma also includes hepatic ultrasound or abdominal CT scan, along with liver function tests, and chest radiography or chest CT scan depending on the medical team's protocol [1]. At the end of this evaluation, the melanoma can be classified according to the TNM system established by the American Joint Committee on Cancer (AJCC), which categorizes uveal melanomas into four stages based on their largest basal diameter and thickness [2].

Some teams recommend performing a biopsy of suspicious lesions, with analysis of genetic alterations [14]. Fine-needle transvitreal cytopuncture (25 G) is the only technique occasionally used, and it carries a very limited risk of tumor cell dissemination [3]. This approach remains controversial and is ultimately proposed only in very rare cases, due to the risk of false-negative results that may provide false reassurance, as well as the significant risk of complications in small lesions, including endophthalmitis, vitreous hemorrhage, and retinal detachment [2].

Management depends on the patient's condition, as well as the tumor's size, location, and metastatic risk. This risk is around 50% at five years, with the liver being involved in more than 80% of cases [1]. Treatment also depends on tumor stage, as defined by the updated TNM classification (Eighth Edition) [2].

Several therapeutic options are available. Targeted radiotherapy is the main therapeutic modality used for the conservative treatment of choroidal melanoma, and two methods can be distinguished [3]: The first is brachytherapy, which consists of contact irradiation using plaque radiotherapy, with either a ruthenium plaque or an iodine-125 disc. The second is proton therapy, which involves irradiation with an accelerated proton beam (60 Gy delivered in four fractions) after surgical localization of the tumor, leading to a slow and progressive reduction in tumor size. This type of irradiation has become widely used and is currently the most commonly employed treatment in Europe for uveal melanomas [15].

Enucleation, with histopathological examination, is indicated for large tumors (thickness greater than 12 mm and diameter greater than 20 mm). It may be supplemented by radiotherapy in cases of extrascleral extension [1]. Surgical removal of the tumor by choroidectomy or partial cyclochoroidectomy is reserved for selected, specific cases. This approach is associated with a high risk of recurrence and a significant rate of perioperative and postoperative complications [4]. Other alternative therapeutic modalities, such as transpupillary thermotherapy and photodynamic therapy, may be proposed but are generally less effective [2].

When metastases are already present at diagnosis, systemic chemotherapy should be started. In this context, priority is given to maintaining the patient's quality of life, and local treatments may therefore be delayed [16].

## Conclusions

Choroidal melanoma is a rare tumor whose management requires a multidisciplinary approach and continued research at all stages of the disease. Tumor size at the time of treatment is an established determinant of metastatic risk, and eligibility for globe-conserving therapy depends largely on tumor dimensions at presentation; earlier detection may therefore widen the therapeutic options available.

The present case illustrates several points of practical value for the general ophthalmologist. First, choroidal melanoma may occur in a very young adult with no identifiable predisposing condition. Second, a peripheral tumor may reach a size precluding globe-conserving therapy while remaining entirely asymptomatic, with preserved visual acuity. Third, multimodal imaging allowed a confident diagnosis and guided the therapeutic decision without recourse to biopsy. Definitive histopathological characterization was not available to the authors, and the significance of the pulmonary nodules remains to be established through ongoing oncological surveillance; no inference regarding this patient's long-term outcome can therefore be drawn from the present report.

These reservations notwithstanding, the case underlines that systematic dilated fundus examination, including careful examination of the peripheral retina, remains the only means of detecting such a lesion in an asymptomatic patient and that recognition of a suspicious pigmented choroidal lesion by the ophthalmologist is the decisive step toward timely management.
